# The Prevalence of Transmitted Resistance to First-Generation Non-Nucleoside Reverse Transcriptase Inhibitors and Its Potential Economic Impact in HIV-Infected Patients

**DOI:** 10.1371/journal.pone.0072784

**Published:** 2013-08-22

**Authors:** Sonya J. Snedecor, Alexandra Khachatryan, Katherine Nedrow, Richard Chambers, Congyu Li, Seema Haider, Jennifer Stephens

**Affiliations:** 1 Pharmerit International, Bethesda, Maryland, United States of America; 2 Pfizer Inc, Groton, Connecticut, United States of America; 3 Pfizer Inc, Collegeville, Pennsylvania, United States of America; University of Pittsburgh, United States of America

## Abstract

Non-nucleoside reverse transcriptase inhibitor (NNRTI)-based highly active antiretroviral therapy (HAART) including efavirenz is recommended as a 1^st^-line treatment choice in international HIV guidelines, and it is one of the most common components of initial therapy. Resistance to 1^st^-generation NNRTIs is found among treated and untreated HIV-infected individuals creating a subpopulation of HIV-infected individuals in whom efavirenz is not fully effective. This analysis reviewed published articles and conference abstracts to examine the prevalence of 1^st^-generation NNRTI resistance in Europe, the United States (US), and Canada and to identify published evidence of the economic consequences of resistance. The reported prevalence of NNRTI resistance was generally higher in US/Canada than in Europe and increased in both regions from their introduction in the late 1990s until the early 2000s. The most recent time-based trends suggest that NNRTI-resistance prevalence may be stable or decreasing. These estimates of resistance may be understated as resistance estimates using ultra-sensitive genotypic testing methods, which identify low-frequency mutations undetected by standard testing methods, showed increased prevalence of resistance by more than two-fold. No studies were identified that explicitly investigated the costs of drug resistance. Rather, most studies reported costs of treatment change, failure, or disease progression. Among those studies, annual HIV medical costs of those infected with HIV increased 1) as CD4 cells decreased, driven in part by hospitalization at lower CD4 cell counts; 2) for treatment changes, and 3) for each virologic failure. The possible erosion of efficacy or of therapy choices through resistance transmission or selection, even when present with low frequency, may become a barrier to the use of 1^st^-generation NNRTIs and the increased costs associated with regimen failure and disease progression underlie the importance of identification of treatment resistance to ensure optimal initial therapy choice and regimen succession.

## Introduction

Resistance to antiretroviral (ARV) drugs is a barrier to effective long-term treatment against human immunodeficiency virus (HIV). With no error-proofing mechanism in its reverse transcriptase protein, the virus is prone to replication errors leading to resistant variants, which are favored under the selective pressure applied by ARV treatment. Common 1^st^-line treatments for HIV include a non-nucleoside reverse transcriptase inhibitor (NNRTI) or protease inhibitor (PI) combined with two or more nucleoside reverse transcriptase inhibitors (NRTIs). Of the NNRTIs, efavirenz (EFV), a 1^st^-generation NNRTI, is a preferred agent recommended by international guidelines [1–3]. EFV was introduced in 1998 and is considered the gold-standard for first line treatment. EFV and other 1^st^-generation NNRTIs (e.g., nevaripine) share common resistance mutations, resulting in extensive cross resistance. Newer NNRTIs with distinct resistance profiles may retain antiviral activity in the presence of these mutations [4,5].

The risk of a major resistance mutation increases by approximately 50% for each year of antiretroviral therapy [6]. Population-wide frequency of 1^st^-generation NNRTI-resistant mutations has intensified in regions with longer histories of NNRTI use and treatment failure (e.g., many North American and Western European countries) [7]. Higher prevalence of resistance in treatment-experienced patients correlates with higher prevalence of transmitted drug resistance (TDR) to newly-infected individuals [8,9]. Novel NNRTI treatment options could reverse this trend, just as initial increases in drug resistance were followed by declines for the NRTI and PI drug classes after introduction of newer, more potent therapies [7].

Given the importance of optimal selection of initial treatment regimens fully active against patients’ viral strains, this targeted literature review sought to examine the epidemiological and economic data relevant to drug resistance by 1) describing the epidemiology of NNRTI resistance in Europe, the US, and Canada in order to understand the size of the patient population for whom initial EFV regimens may not be appropriate and 2) identifying published economic data presenting the costs associated with drug resistance.

## Literature Search Methods

PubMed/MEDLINE and EMBASE were searched to identify literature reporting the epidemiology of NNRTI class resistance and the economic burden associated with resistance or treatment failure in Europe, the US, and/or Canada. These searches were limited to results published in English, years 1996 to 2011, and reported from studies of human subjects. Additionally, relevant abstracts presented at scientific conferences were identified through EMBASE and internet searches.

## Results

### 1^st^-generation NNRTI resistance in treatment-naïve individuals TDR

**Figure 1:**
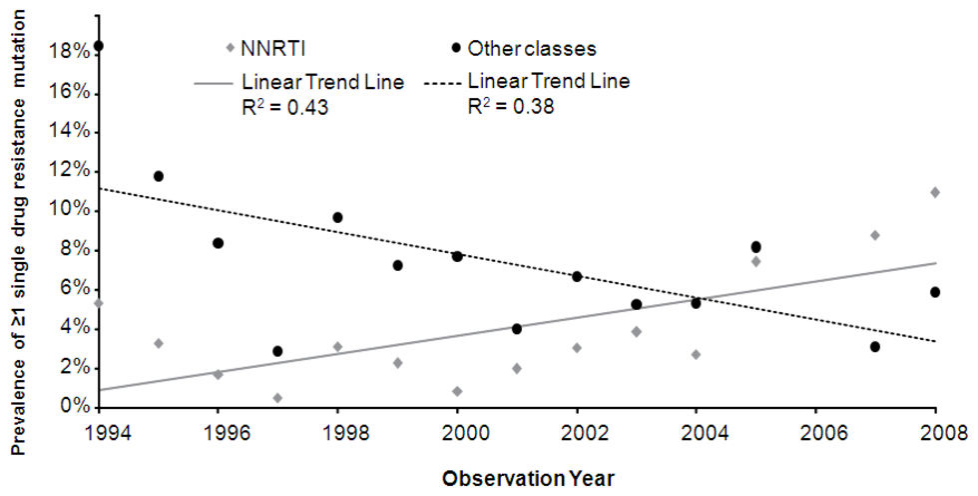
NNRTI and other class resistance in treatment-naïve patients in North America and Western Europe [12,13].

In the transmission of viral strains containing resistance mutations, the infecting individuals are patients who have acquired resistance as a result of exposure to ARVs or are treatment-naïve persons who themselves have been infected with resistant virus [10,11]. Stanford University’s online data repository of NNRTI and other drug class resistance data in treatment-naïve individuals [12,13] is perhaps the most robust general source of NNRTI resistance information publicly available. These data, aggregated from studies of patients in North American and Western European countries, illustrate an increasing prevalence trend of transmitted NNRTI resistance through 2008 (Figure 1).

**Figure 2:**
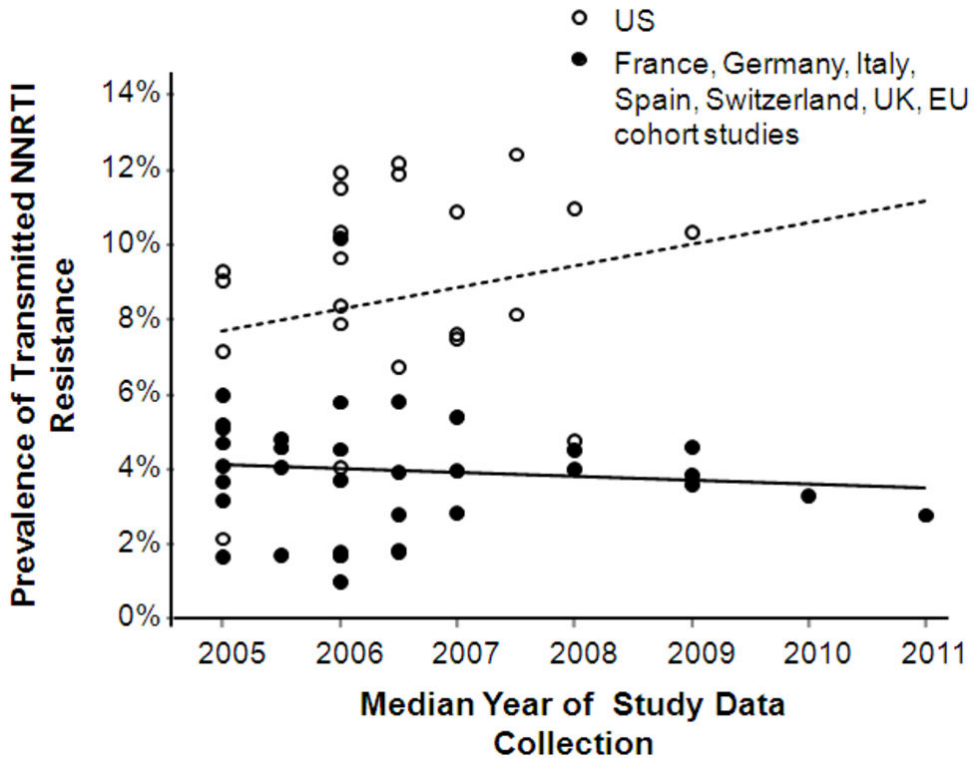
Reported prevalence of 1^st^-generation NNRTI resistance in the US and European countries among treatment-naïve patients. Note: Solid and dotted lines indicate trend lines for Europe and US, respectively. References available from the author.

After the introduction of 2^nd^-generation NNRTIs and other new ARVs in 2008, the previously upward trend in transmitted NNRTI resistance in treatment naïve-patients appears to have stabilized or decreased at least in some European countries (Figure 2). In the US, recent data in a study of those in early HIV infection showed a significant increase in transmitted NNRTI resistance from 7% to 15% (p=0.04) from 2003–2007 and a significant drop in all-class TDR in 2008, which the authors attributed to increasing virologic suppression of drug-resistant patients using newly-licensed ARVs [14]. However, recent data from several US studies collectively showed an increasing trend in the prevalence of 1^st^-generation NNRTI resistance [15] (Figure 2).

In Canada, declines in NNRTI resistance were noted in treatment- naïve and treatment-experienced patients, from 10% in 2000 to 5% in 2008 and 35% in 2000 to 15% in 2009, respectively. No further declines were observed in years 2010-2011 for NNRTIs [16]. However, another Canadian study found that despite a decline in the major NNRTI resistance mutations K103N, Y181C, and G190A among patients failing treatment (from 36.7% in 2002 to 19.8% in 2009), the prevalence of these mutations in treatment-naïve patients remained stable [17].

In Europe, similar trends of TDR and NNRTI resistance were observed over time. The United Kingdom (UK) Collaborative Group on HIV Drug Resistance (2012) observed that prevalence of NNRTI resistance remained stable around 3.6% from 2002 to 2009. In Switzerland, transmitted NNRTI resistance increased from 0% in 1997 to 4.5% in 2007, also remaining stable in recent years (2007-2010) in newly-infected patients [18].

### Correlates of transmitted resistance

The prevalence of TDR correlates with the amount of virus circulating in the population. A seven-year Spanish surveillance study confirmed that patients with unsuppressed viral replication contribute to TDR [19]. This surveillance study reported that the prevalence of TDR in persons with acute HIV-1 seroconversion increased with greater proportions of chronically-infected individuals with detectable circulating plasma HIV-1 RNA in the population. The Swiss HIV Cohort Study also observed that the proportion of newly-infected patients with TDR tended to be associated with the mean viral load of the total HIV population of the previous year [18].

No clear correlation between socio-economic risk factors and the presence of acquired drug resistance or transmitted drug resistance emerged from the literature review except those that are proxies for ARV exposure, such as insurance status and ART adherence. Some studies noted associations between age or sex and TDR [20–22]. However, other studies found no association between resistance and these factors or sexual identity/HIV risk group, duration of infection, recent drug injection use, homelessness, prior incarceration, or education [6,11,23,24]. One study reported that “other or mixed race” and health insurance status were significantly associated with the likelihood of having at least one major mutation, but insurance status was correlated with having received prior ARV therapy [6]. Other reviewed studies did not find insurance status to be associated with resistance [6,25].

Some resistance prevalence studies have observed that those infected with non-B HIV subtypes had lower rates of TDR, which has led some researchers to suggest that infection with non-B subtype viral strains may be protective against the development of resistance mutations; thus explaining the lower prevalence of resistance mutations in these patients [22,26–28]. For example, a prospective Belgian study specifically assessing the prevalence, epidemiology, and risk factors of drug resistance in newly-diagnosed individuals reported that the prevalence of resistance was lower for those infected with non-B subtypes. However, when the authors restricted the analyses to include only individuals infected in Belgium, no association between subtype and resistance was found [29]. Similar findings were observed in an analysis of patients residing in the UK wherein having been born in the UK (but not ethnicity or viral subtype) was the most robust predictor of resistance mutations in multivariate analysis (relative risk = 0.10, p=0.002) [26].

These findings suggest that the effect of subtype B on the presence or absence of transmitted resistance may not be attributable to specific viral characteristics of the subtype, but rather to a combination of other risk factors such as population-level use of ARVs at the time and locality of infection. That is, those carrying non-B subtypes in the aforementioned studies originated largely from African countries where HAART was introduced later than in Europe and may have been less widely available. Thus, during these patients’ time of infection, lower levels of population-wide ARV exposure likely translated to lower levels of population-wide drug resistance and TDR, regardless of subtype [7].

### Persistence of NNRTI resistance mutations

Transmitted NNRTI-resistant mutations have the ability to persist for years after initial infection, and acquired resistance due to drug exposure can persist beyond NNRTI treatment termination. For example, Joly et al. [30] reported significant declines in NNRTI resistance mutations one year after treatment discontinuation due to virologic failure. The authors also found that 70% of these patients harbored NNRTI-resistant variants at one year follow-up despite switching to a non-NNRTI regimen. This suggests that, although levels of drug-resistant variants declined after patients discontinued therapy, the drug-resistant variants that remained persisted, an indication of the low impact of NNRTI resistance mutations on viral fitness [30,31]. These findings have clinical significance in that transmitted NNRTI resistance potentially limits future NNRTI treatment options for the remainder of a patient’s lifetime.

### Low-frequency NNRTI variants

NNRTI-resistant variants existing with frequency less than 20% of a patient’s viral population – often referred to as “low-frequency” mutations – are not generally detected by standard genotype testing methods [23,32,33], potentially underestimating the prevalence of ARV resistance. Several studies showed high-sensitivity assays detected resistance-associated mutations in twice as many patients compared to standard sequencing methods [26,34–37]. Low-frequency NNRTI-resistant variants were also detected in NNRTI-experienced patients and were associated with reduced virologic response to EFV-containing multidrug regimens [38].

If the presence of these low-frequency mutations impairs treatment effectiveness, they may present a clinical challenge to physicians when choosing optimal first-line treatment since ultra-sensitive testing methods are not used in routine clinical practice. To understand the clinical impact of these mutations, Li et al. [39] conducted a meta-analysis to assess the association of preexisting drug-resistant, low-frequency variants with the risk of NNRTI-based ARV failure in ART-naïve participants initiating NNRTI-based regimens. The authors performed a 10-study pooled analysis of 985 patients to estimate the differential risk of treatment failure in patients with low-frequency resistance mutations compared to those without. The authors found low-frequency mutations were associated with an increased risk of virologic failure (HR=2.3 [95% CI, 1.7-3.3]), even after controlling for medication adherence [40], race/ethnicity, baseline CD4 cell count, and plasma HIV-1 RNA levels. Specifically, the risk of virologic failure in patients with low-frequency NNRTI-resistant variants was higher than the risk of failure in patients with low-frequency NRTI resistance (HR=2.6 [95% CI, 1.9-3.5] versus 1.6 [0.1-17.7], respectively). Li et al. also found a dose-dependent increased risk of virologic failure in those patients with 0, 1-9, 10-99, 100-999, or ≥1000 copies of low-frequency variants per ml of plasma. This finding was also confirmed by analyses of nevirapine treatment in women previously treated with a single dose to prevent mother-to-child transmission at childbirth [41,42].

Other recent studies of the clinical impact of low-frequency mutations in treatment-naïve patients initiating therapy have reported mixed outcomes. Some studies have shown increased probability of virologic failure or increased time to virologic suppression in patients with low-frequency mutations [43–47], whereas others have not [48–50]. Although there is still debate within this field, these outcomes appear to suggest that only certain NNRTI mutations may be clinically relevant when present at low frequency (i.e., K103N, Y181C) and these mutations may not impair efficacy of newer, 2^nd^-generation NNRTIs [51,52].

### Health economic burden of HIV

No studies reporting healthcare costs associated with drug resistance were identified. The majority of economic studies described lifetime costs of HIV, costs associated with different stages of disease (i.e., different CD4 cell levels), and costs due to treatment failure or treatment changes. Studies of the latter could potentially be extrapolated to represent the costs of resistance, considering that treatment failure and switching may follow from the development of drug resistance or selection of a treatment regimen to which a patient harbored resistance. This section presents, all costs identified.

### Lifetime healthcare costs of HIV-infected patients

ART has been effective in extending life expectancy among patients with HIV; however, lifetime costs of HIV still represent a significant economic burden. Schackman et al. [53] projected the lifetime costs of HIV in the US from time of initial care (with <350 CD4 cells/µl) until death to be $618,900, or $2100 per month (2004 USD). The majority of these costs (73%) were attributable to ARV drugs. More recently, Sloan et al. [54] projected the lifetime cost to be €535,000 per patient (2010 EUR) among HIV patients in northern France initiating treatment with a CD4 level of 372 cells/µl. In both studies, monthly and annual costs increased as patients’ CD4 cell count decreased or stage of HIV disease progressed.

Initiation of HIV treatment in later stages of disease has been found to be an important determinant of life expectancy and higher annual costs of care driven by greater inpatient treatment utilization (Table 1). In the Sloan et al. study, annual costs were greater among patients initiating care with CD4 levels <50 cells/µl, an indicator of more severe disease, compared to those with >500 cells/µl (€36,540 and €19,240, respectively [2010 EUR]) with inpatient costs driving this difference (38% and 4% due to inpatient costs, respectively) [54]. In the Shackman et al. study, a patient initiating care with ≤50 CD4 cells/µl was estimated to have more than double the monthly costs than those with CD4 >300 cells/µl ($4700 vs. $2000 [2004 USD], respectively) where inpatient costs accounted for a larger proportion than ARV therapy costs (49% vs. 10%, respectively) [53]. Furthermore, Canadian patients initiating care with <350 CD4 cells/μl had significantly higher costs, with direct medical costs almost twice that of patients initiating with >350 CD4 cells/µl (Table 1) [55]. This trend suggests that efforts to identify HIV patients and initiate treatment in earlier disease stages may help to curb the payer burden of health care resource utilization such as hospitalizations and the humanistic burden of HIV-related morbidity and mortality. From a societal perspective, earlier treatment has also been shown to be cost-effective given that the added life-years are achieved with minimal increases in lifetime costs [56].

**Table 1:** Differential healthcare costs of initiating treatment at various CD4 cell strata.

**France (2010€, N=1775 patients)** [54]				
**CD4 cell stratum**	**Annual**	**Lifetime**	**Components of lifetime cost**	**Life expectancy**
**>500**	19,240€	534,800€	ARV (81%), Day care (11%), Inpatient (4%), Outpatient and lab (4%)	27.5 years
**351-500**	15,970€**	NR	ARV (70%), Day care (15%), Inpatient (10%), Outpatient and lab (5%)	NR
**201-350**	22,500€	NR	ARV (64%), Day Care (13%), Inpatient (17%), Outpatient and lab (6%)	NR
**101-200**	28,000€	NR	ARV (51%), Day Care (16%), Inpatient (24%), Outpatient and lab (8%)	NR
**51-100**	30,000€	513,200€	ARV (49%), Day care (15%) Inpatient (26%), Outpatient and lab (10%)	23.8 years
**<50**	36,540€	NR	ARV (41%), Day Care (11%), Inpatient (38%), Outpatient and lab (9%)	NR
**Canada (2009 CAD, N=193 patients)** [55]				
**CD4 cell stratum**	**Monthly (± SD)**	**Annual (± SD)**	**Components of cost**	
**>350**	$914 ± $452	$10,968 ± $5,677	Direct HIV (32%), HIV Drugs (30%), Outpatient (39%), HIV-related inpatient (85), Non-HIV inpatient (49%)	
**<350**	$1419 ± $378	$17,028 ± $5,031	Direct HIV (68%), HIV Drugs (70%), Outpatient (61%), HIV-related inpatient (92), Non-HIV inpatient (51%)	
**United States (2004 USD, N=59,093 patient-months)** [53]				
**CD4 cell stratum**	**Monthly**	**Lifetime**	**Components of cost**	**Life expectancy**
**<350**	$2100	$618,900	ARV (73%), Inpatient (13%), Outpatient (9%), Other HIV-related medication and laboratory (5%)	24.2 years
**<200**	$2500	$567,000	ART (58%), Inpatient (21%), Outpatient (10%), Other HIV-related medication and laboratory (11%)	22.5 years

* Study conducted over 15-year period; ** The CD4 strata of 351-500 cells had a lower proportion of patients being treated with ARVs (70%) than the >500 strata (81%), contributing to the lower relative cost.

NR = not reported?

### Costs of disease progression

**Figure 3:**
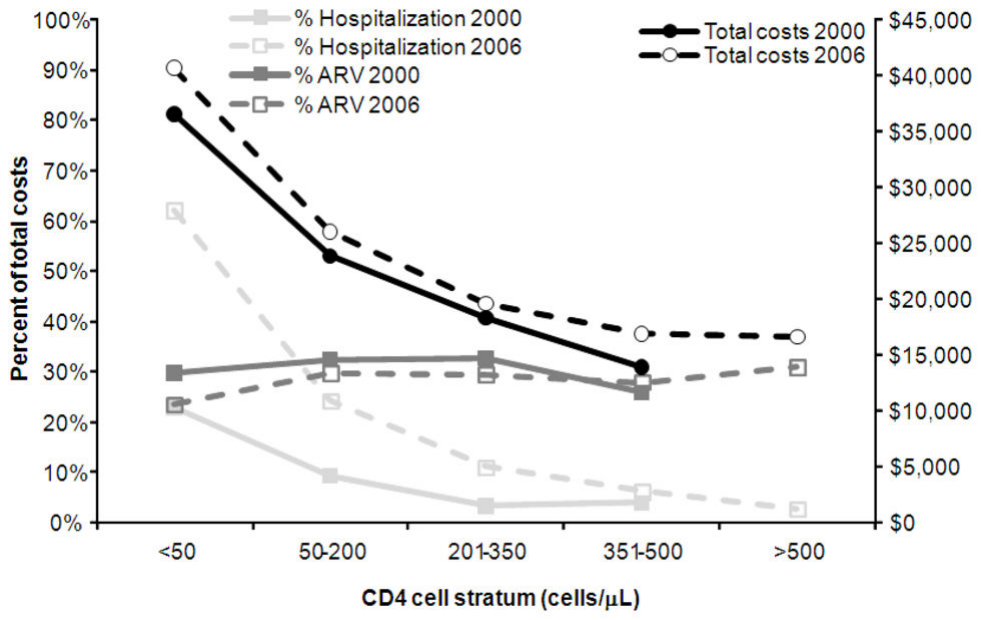
US total healthcare costs of patients with HIV. Note: Right axis: mean per patient costs in 2000 [57] and 2006 [58]; Left axis: proportion of total healthcare costs due to hospitalizations and ARV drugs.

The higher monthly cost of care among treated or untreated HIV patients with more advanced disease is well-documented. Two US studies reported similar total annual costs of patients in several CD4 cell strata for the years 2000 [57] and 2006 [58], respectively (Figure 3). From 2000 to 2006, the proportion of total annual healthcare costs attributable to ARV costs remained constant, while the proportion of costs due to hospitalizations nearly tripled from 24% to 62% for patients in the lowest CD4 cell stratum (<50 cells/µl) (Figure 3). The reported total annual cost in 2006 was $20,000 (USD), similar to the range of results presented in a 2010 study by Hellinger and Encinosa (2010) [59] ($16,700-$21,000; 2005 USD).

Data from northern Italy over the years 2004-2007 showed significantly greater annual costs for CD4 levels <200 cells/µl (€12,700, p=0.01 [2008 EUR]), versus patients with CD4 counts between 200–499 (€9600) or >500 cells/µl (€9450) [60]. In Germany, the annual cost difference between patients with >500 cells/µl and those <200 cells/µl was €10,200 (2008 EUR) in annual costs [61]. When comparing patients in the UK initiating treatment on first-line therapy, higher annual costs occur among those with <200 CD4 cells/μl (£12,800 [2008 GBP]) compared to those with >200 cells/µl (£10,500). This difference was attributable in part to two-fold higher inpatient costs (£1,500 vs. £700, respectively) [62].

### Healthcare costs by virologic failure and subsequent succession of treatment regimens

**Figure 4:**
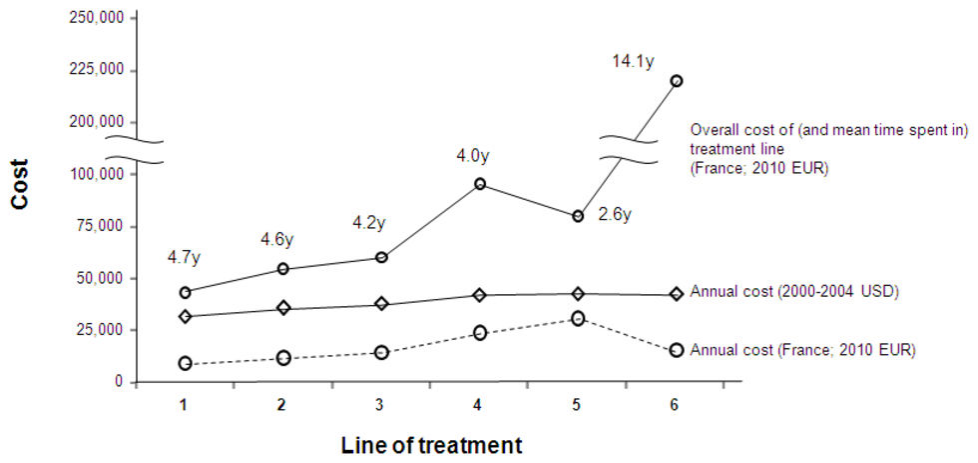
Mean per-patient healthcare costs for patients on increasing lines of treatment in US [63] and Europe [54].

Overall healthcare costs also increase with subsequent lines of treatment. One report demonstrated total annual costs ranged from $31,700 for a patient on a 1^st^-line HAART regimen to $42,600 on 6^th^-line (2000-2004 USD) [63] (Figure 4). These costs increased by a mean of $3,400 per patient in each subsequent line of treatment. In a separate study presented in 2010, total mean healthcare costs estimated over a follow-up period of up to 60 months were $35,000 higher for patients on a 3^rd^- or greater line treatment regimen compared to patients on a 1^st^- or 2^nd^-line treatment regimen (USD, cost year not reported) [64].

**Figure 5:**
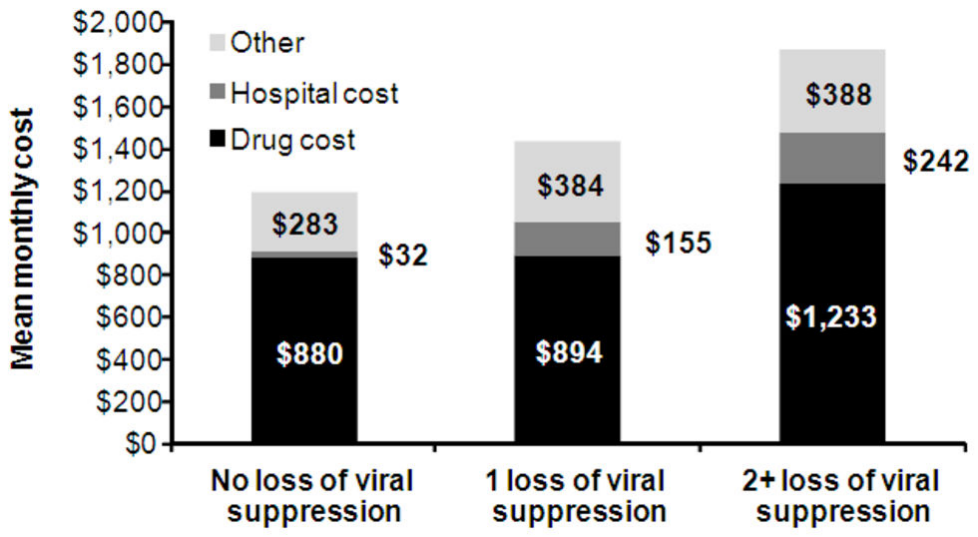
Mean monthly per patient healthcare costs of US patients on ART from 1996 to 1998 [65].

Each episode of virologic failure reduces the number of available future treatment options, resulting in higher likelihood of progression to AIDS and increased health care costs. In a longitudinal assessment using personal interviews and medical and billing records of 743 patients with HIV, monthly healthcare costs were found to be higher in patients who experienced treatment failure than in those who did not [65] (Figure 5). Moreover, per patient total monthly healthcare costs increased for each additional loss of viral suppression with monthly costs of $1400 and $1900 for 1 and ≥2 loss(es) of viral suppression, respectively (USD, cost year not reported). Recent estimates of the marginal healthcare costs associated with increasing line of treatment or virologic failure were not identified.

## Discussion

While EFV is the preferred NNRTI component of ARV combination therapy for HIV treatment, the prevalence of EFV (and other 1^st^-generation NNRTIs) resistance has increased over the years and remains endemic in many regions. Newer NNRTI treatments provide expanded access to effective therapies for patients who have transmitted or acquired 1^st^-generation NNRTI resistance [66]. These newer treatments may have particular importance as new advances in high-sensitivity genotypic testing allow researchers to identify patients harboring low-frequency resistant viruses who have increased likelihood of virologic failure [39], including patients in whom EFV is a sub-optimal 1^st^-line choice. Accurate identification of patients infected with resistant virus will be necessary for clinicians to assign fully-effective treatment regimens and reduce the risk of virologic failure and its associated costs [67].

No publications investigating the cost of ARV resistance were identified, but rather most identified cost studies reported costs of disease progression and/or treatment failure. This gap in the literature is likely attributable to data availability and ease of cost identification associated with these events as patients’ CD4 cell levels are monitored regularly and treatment switching can be detected via medical claims databases. Differentiating costs of treatment switching specifically due to resistance, as compared to switching due to other factors such as intolerance or adherence, would require knowledge of genotypic testing results at the time of treatment switch, which may be difficult to obtain. Similarly, differential costs in patients with and without TDR who begin treatment would require genotypic testing results prior to therapy initiation, which is not generally available to economic researchers. The increasing costs associated with regimen failure and disease progression currently reported in the published HIV economic literature provide the closest proxies to costs associated with drug resistance and underscore the importance of ensuring optimal initial therapy choices and regimen succession.

In addition to the lack of data on the costs of resistance, additional cost data gaps were identified. Several studies documented increasing healthcare costs resulting from HIV disease progression and treatment changes, but none examined these costs adjusted for the possibility of associated decreases in CD4 cells, which could confound the estimates. Also, this review found a notable lack of published studies using more recent healthcare cost data in the US.

In light of the clinical consequences of ARV resistance, new studies are needed to measure healthcare costs incurred after treatment change among patients switching due to resistance versus those switching for other reasons (for example, identified via medical chart review and physician interviews) and adjusted for CD4 cell level. Without these studies, it is impossible to understand the economic consequences of virologic failure apart from those of disease progression and to definitively assess the down-stream clinical and economic impact of resistance.
